# The Limits of Acute Anemia

**DOI:** 10.3390/jcm11185279

**Published:** 2022-09-07

**Authors:** Tina Tomić Mahečić, Roxane Brooks, Matthias Noitz, Ignacio Sarmiento, Robert Baronica, Jens Meier

**Affiliations:** 1 Department of Anesthesiology and Intensive Care Medicine, University Hospital Center Zagreb—“Rebro”, 10000 Zagreb, Croatia; 2 Department of Anesthesiology and Critical Care Medicine, Kepler University Hospital GmbH, Johannes Kepler University, 4040 Linz, Austria; 3 Department of Anesthesiology, Clinica Santa Maria, Santiago 7520378, Chile

**Keywords:** anemia, transfusion threshold, patient blood management

## Abstract

For many years, physicians’ approach to the transfusion of allogeneic red blood cells (RBC) was not individualized. It was accepted that a hemoglobin concentration (Hb) of less than 10 g/dL was a general transfusion threshold and the majority of patients were transfused immediately. In recent years, there has been increasing evidence that even significantly lower hemoglobin concentrations can be survived in the short term without sequelae. This somehow contradicts the observation that moderate or mild anemia is associated with relevant long-term morbidity and mortality. To resolve this apparent contradiction, it must be recognized that we have to avoid acute anemia or treat it by alternative methods. The aim of this article is to describe the physiological limits of acute anemia, match these considerations with clinical realities, and then present “patient blood management” (PBM) as the therapeutic concept that can prevent both anemia and unnecessary transfusion of RBC concentrates in a clinical context, especially in Intensive Care Units (ICU). This treatment concept may prove to be the key to high-quality patient care in the ICU setting in the future.

## 1. Introduction

The loss or deficient production of erythrocytes leads to anemia, defined as a reduction in erythrocytes and Hb concentration in the blood [1]. Due to many possible causes, anemia is widespread and is therefore a global burden of disease [2]. However, the phenomenon of “acute anemia” has become more common in the last century. In contrast to chronic anemia, acute anemia arises in most cases when acute blood losses are substituted by acellular solutions. In acute hemorrhage, primarily erythrocytes and plasma are lost simultaneously, which keeps the concentration of erythrocytes in the remaining circulating blood constant. The subsequent transfer of interstitial fluid into the intravascular space leads to a reduction in the erythrocyte concentration and, consequently, to a drop in the hemoglobin concentration and the hematocrit values [3]. Blood losses are also often replaced with acellular solutions, such as crystalloids, or to a much lesser extent with colloids, which leads to an immediate reduction in the erythrocyte concentration and thus to dilutional anemia [4]. The German physiologist Kronecker was the first who demonstrated in animal experiments that extensive, acute blood loss can be survived even at very low hemoglobin concentrations if the lost volume is replaced with water or salted solutions [5]. He thus proved that acute hypovolemia is significantly more dangerous for the animals’ integrity than dilutional anemia caused by volume administration, as long as a certain number of circulating erythrocytes remain [6]. It is now known from many animal experiments that the lowest physiological short-term limit of acute anemia is about 3 g/dL, depending on the physiological compensation reserve and the clinical situation [7,8,9,10,11]. However, individual clinical case reports suggest that patients with significantly lower Hb values survived without long-term sequelae, with the lowest documented value being 0.7 g/dL [12].

The fact that milder forms of anemia also have a considerable influence on physical performance was discovered in 1946 by the gynecologist Adams [13]. He proved that postpartum women were physically more resilient if their Hb was above 10 g/dL (hematocrit > 30%). This led to the so-called “10/30 rule”, which resulted in a Hb of 10 g/dL being held as the typical clinical threshold for the transfusion of RBC for many years. This approach was established as a standard in everyday clinical practice [14].

In 1999, for the first time, it was proven in a randomized controlled trial that even in intensive care patients, a Hb of 7 g/dL has a similar outcome compared to patients with a Hb above 9 g/dL [15]. This study indicates that a Hb of 10 g/dL should probably not be the limit for acute anemia. In addition, case reports from Jehovah’s Witnesses provide increasing evidence that patients with significantly lower Hb values may survive without any sequelae. This opens the question of why a Hb of 10 g/dL does not represent the tolerable short-term limit for acute anemia. In contrast, it might have a long-term influence on morbidity and mortality [16]. However, an increasing amount of the literature questions the scientific approach of comparing liberal versus restrictive transfusion regimes [17,18,19]. Therefore, this narrative review aims to enlighten the limits of acute anemia from a physiological, clinical, and “patient blood management” (PBM) point of view, highlighting the effects of therapeutic alternatives. We did not perform a systematic review with a systematic literature search but combined the most pivotal literature from animal experiments and clinical trials.

## 2. The Physiological Point of View

One of the most important tasks of blood circulation is transporting oxygen to the cells of individual tissues [20]. Since oxygen dissolves very poorly in blood plasma, adequate oxygen supply depends on the presence of erythrocytes, which uniquely bind oxygen. The sigmoidal oxygen dissociation curve of hemoglobin ensures oxygen uptake in the lung and oxygen release in the tissues [20]. Therefore, the release and uptake of oxygen by erythrocytes is a passive process that primarily depends on the microenvironment and is significantly altered in septic patients [21]. Typical venous oxygen saturation in tissues is 65%, which means that only about one-third of the transported oxygen is released to the tissues. At least 2/3 recirculates back to the right heart via the venous circulatory system [20].

**Figure 1:**
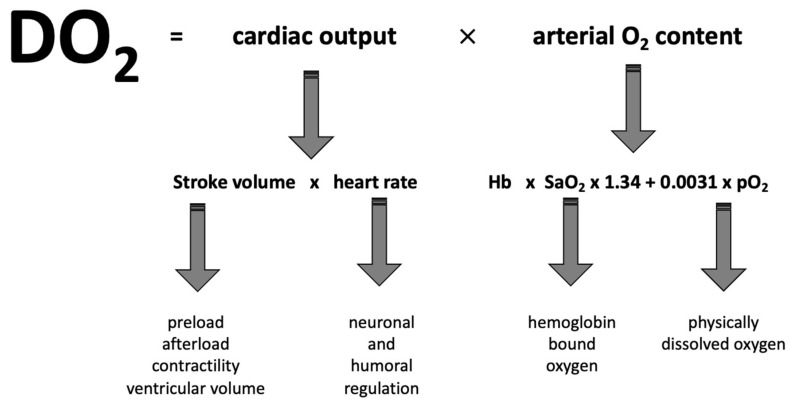
Oxygen delivery (DO_2_) is defined as the product of cardiac output and arterial oxygen content (CaO_2_). Cardiac output is defined as the product of stroke volume and heart rate. Stroke volume depends on preload, afterload, contractility, and ventricular volume, whereby neuronal and humoral factors modulate heart rate. Arterial oxygen content is defined as the sum of hemoglobin-bound oxygen (hemoglobin concentration times arterial saturation times Hüfner’s number) and physically dissolved oxygen (arterial oxygen partial pressure times a constant).

The absolute amount of transported oxygen also depends linearly on the cardiac output. Control mechanisms of cardiac output are complex; they include heart rate (which is, on the one hand, controlled by the autonomic nervous system and, on the other hand, controlled by humoral regulation) and stroke volume (Figure 1). The stroke volume is determined by the wall shear stress of ventricles (preload), myocardial contractility, and peripheral vascular resistance (afterload) [22]. The vascular resistance, in particular, depends on the blood’s viscosity. The higher the Hb value, the more peripheral vasoconstriction occurs via shear forces on the endothelium. In acute anemia, these shear forces decrease, and the peripheral resistance drops—reflectively, the cardiac output and thus the oxygen supply to the tissues increase [23]. In awake patients, acute dilutional anemia also increases cardiac output by increasing heart rate [24], an adaption mechanism that typically does not occur in anesthetized individuals. Since neural or humoral pathways do not directly regulate the oxygen delivery in the organism, the relative increase in cardiac output exceeds the relative decrease in the Hb value. Thus, the oxygen supply to the tissues paradoxically increases. With the further decline in Hb, oxygen delivery reaches its baseline level [25]. Only at very low Hb values (3 g/dL) can the organism’s oxygen requirements no longer be met, and tissue hypoxia occurs.

All these mechanisms are necessary to meet the oxygen requirements of the organs and tissues. In the literature, a value of 3.6 mL/min/kg for an adult is often given for the resting oxygen demand. However, this value can vary for individual organs, from 2 mL/min/kg for the skin to 100 mL/min/kg for the myocardium. Therefore, different organs react quite differently to an acute restriction of the oxygen supply. Furthermore, it cannot be predicted whether an individual patient can increase cardiac output to such an extent for a given Hb that the limits for oxygen demand can be met. 

Theoretical deduction of the limit of acute anemia is impossible. The complexity and individuality of the potential compensatory mechanisms prevent adequate prediction of the outermost limit of acute anemia. Furthermore, it is difficult to define what determines the limit of anemia in a specific setting. Theoretically, several different outcome parameters such as short-term survival, long-term survival, lactate concentration, pH, tissue oxygenation, etc., can be used to define the limit of anemia. For example, if a change in long-term survival is used to define the limit of anemia, this might be completely different from a situation where an increase in lactate is used to define the limit of anemia. How this influences the definition of the limit of anemia will be discussed later in the article. 

### 2.1. What Is Known from Animal Experiments?

From a theoretical point of view, the most solid outcome parameter of acute anemia is short-term survival. Several animal experiments have been performed that investigated the outermost limit of anemia in terms of oxygen transport and tissue oxygenation and their influence on short-term survival. However, the first systematic investigations focused on oxygen transport and tissue oxygenation. van Bommel and coworkers were one of the first to determine the hemoglobin concentration in anesthetized pigs, where the compensatory mechanisms of acute anemia were unable to stabilize oxygen consumption (VO_2_) [26]. The main finding of this study was that the systemic VO_2_, the cerebral µpO_2_, and the intestinal mucosal µpO_2_ became impaired at the same stage during hemodilution. In contrast, the intestinal serosal µpO_2_ became impaired at an earlier stage. In this model, the decline in VO_2_ was interpreted as supply dependency; therefore, a hematocrit of 7.6% was defined as the outermost limit of acute anemia. 

Torres Filho and coworkers performed similar experiments in rats [27]. They could show that, until a hemoglobin concentration of 6 g/dL was reached, oxygen transport and tissue oxygenation were barely influenced, but at a hemoglobin concentration of 3 g/dL, rats’ compensatory mechanisms were incapable of avoiding tissue hypoxia due to an overcritical reduction in tissue oxygenation. Similar values could also be found in large animal models, where a hemoglobin concentration close to 3 g/dL has been demonstrated to be the limit of the compensatory mechanisms of acute anemia, resulting in severe short-term mortality [7,28,29]. 

However, this physiological approach is subject to some relevant limitations. For example, it assumes a critical limit in the oxygen supply to the tissues in all organs simultaneously, although this is not the case. Several animal studies have demonstrated that the anemia tolerance of individual organs can differ [9,10,27]. Initially, it was erroneously assumed that the myocardium, as the carrier of the compensatory mechanisms of acute anemia, should determine the limit of anemia tolerance of the whole body [24]. In contrast to this assumption, it has been demonstrated that the kidneys, for example, become hypoxic when the compensatory mechanisms of acute anemia still maintain the integrity of the cardiovascular system [9,11]. In addition, the physiological principles described above just apply if the lost blood volume is replaced to maintain normovolemia. Only in this case is there no release of endogenous catecholamines during anemia [7]. In the case of hypovolemia, peripheral vasodilation, one of the main compensatory mechanisms of acute anemia, is counteracted. This prevents the combination of acute anemia and acute hypovolemia from being compensated as well as acute anemia alone [30]. Lastly, none of these animal studies investigated the long-term outcome of such severe anemia. Therefore, this outermost limit of anemia cannot be considered safe for a longer time.

### 2.2. What Is Known about Extreme Anemia in Humans?

#### Outcome Short-Term Survival

The considerations mentioned above for animal models are supported by several anecdotal case reports in humans [12,31,32] and correlate with studies in patients for whom blood is not an option (mostly Jehovah’s Witnesses), who refuse transfusion of allogeneic blood for personal or religious reasons. Transfusion in this cohort not only would disregard patient autonomy but can also result in medicolegal problems [33]. Several major case series correlate the lowest perioperative Hb concentration with survival [16,34,35,36]. They showed that patients could survive with a Hb value of ≈3 g/dL for a short period. Still, these studies also clearly demonstrated that if extreme anemia is not corrected, mortality below a Hb value of 6 g/dL notably increases. This is in contrast to the theoretical physiological considerations mentioned above. Regarding oxygen transport and tissue oxygenation, the limit for acute anemia should be at much lower hemoglobin concentrations than 6 g/dL. This phenomenon can therefore be seen as a reference that a low threshold for transfusion cannot solely be defined based on purely physiological considerations. It is difficult to assess whether the temporal component is the only reason for this so that short-term extreme anemia can be better tolerated than moderate anemia of longer duration.

It is sometimes recommended to use physiological transfusion triggers rather than fixed Hb values for the transfusion of RBC concentrates. These may theoretically indicate a critical limitation in oxygen transport and tissue oxygenation. Although very interesting from a physiological point of view, extensive clinical use cannot be recommended at present [37].

Summarizing these results, it can be concluded that acute anemia below a Hb of 6 g/dL is not safe as a transfusion threshold in everyday clinical practice since the risk of increased mortality is unacceptable for ICU patients. However, which hemoglobin concentration above 6 g/dL can be considered safe without transfusion is still open for discussion. Furthermore, even transfusions deemed appropriate due to a low hemoglobin concentration could be avoided if adequate measures were taken in advance [38]. The benefit/risk ratio of transfusion at a specific threshold might depend on the individual clinical situation and the individual capability of a patient to compensate for the reduction in the hemoglobin concentration. For this reason, numerous studies have been conducted in different patient populations to investigate the mortality and morbidity of acute anemia with or without transfusion.

## 3. The Clinical Point of View—The Effect of Acute Anemia and Transfusion on Morbidity and Mortality

In daily clinical practice, it is impossible to study the morbidity of acute anemia without the influence of transfusion. A potential clinical study that includes a control group with extensive anemia over a prolonged time might be considered unethical. Therefore, most clinical studies compare groups of patients subjected to a liberal or a restrictive transfusion regime [39]. These clinical studies investigate both regimes in patients with different pathologies and cannot be easily compared to each other. Typically, the effects of prolonged acute anemia with eventual transfusion at a lower threshold (in these studies, often called a restrictive transfusion regime) are compared with the results of transfusion of allogeneic blood after a short period of acute anemia (liberal transfusion regime) [40]. Although this reflects the typical clinical approach, such studies cannot describe the safe lower limit of acute anemia for oxygen transport but can only provide valuable guidance on the most useful clinical practice in terms of therapeutic modalities, a point of view that will be discussed later in the article.

A typical study considering this restriction was performed by von Heymann and coworkers in cardiac surgery patients [41]. They demonstrated that perioperative anemia is an independent risk factor for mortality and that this risk is increased by transfusion of RBC concentrates. Furthermore, the risk of dying is correlated with the degree of anemia: the more anemic the patients were, the higher their probability of death. These results were later confirmed by Jabagi in a similar study [42]. He also showed that perioperative anemia is an independent risk factor for perioperative death and that this risk is additionally increased by the transfusion of RBC concentrates. 

In addition to these findings, preoperative anemia is independently correlated to perioperative mortality. This has been shown many times in large patient cohorts. Most impressive in this context is a study by Musallam from 2011 in which he demonstrated in 227,425 patients that even mild to moderate anemia increases the perioperative risk of death by more than 20% [43]. Mild to moderate anemia was defined as a Hb value of 10–13 g/dL, which is far from limiting oxygen transport and tissue oxygenation. Thus, the reason for the increase in morbidity and mortality cannot be sought in inadequate tissue oxygenation but is likely to have other causes that cannot be deduced from the original study.

These results were repeated several times for surgical patients, showing a statistical correlation between mild anemia and a negative perioperative outcome [44,45,46]. Unfortunately, all these studies have to be seen as descriptive, and the association between anemia and perioperative mortality cannot be proven causally due to this fact. Theoretically, one or more confounders could exist that represent the actual reason for increased perioperative mortality. In this case, perioperative anemia could only be linked statistically to these confounders. 

Hebert conducted the first and perhaps the most significant study comparing a liberal with a restrictive transfusion regime in intensive care patients [15]. He demonstrated that a transfusion trigger of 7 g/dL was not associated with higher mortality compared to a transfusion trigger of 9 g/dL. At that time, this new approach opened the field for this point of view. Consequently, similar studies were performed in various patient cohorts, and in the vast majority of cases, a restrictive transfusion regimen was non-inferior to a liberal one [47]. However, as demonstrated by Carson et al. in this Cochrane review, there are “insufficient data to inform the safety of transfusion policies in certain clinical subgroups”, which include acute coronary syndrome, myocardial infarction, traumatic brain injuries, cancer including hematological malignancies, and stroke, which represents a large percentage of the population of patients in hospitals [48,49,50].

Although all of these studies demonstrated that a “restrictive” transfusion regime is as safe as a “liberal” one, no study can show whether other thresholds might be safer than those investigated. In this context, “safe” always has to be seen as connected to the specific study and the two groups compared.

Furthermore, many patients treated in an ICU have comorbidities that are not present in other settings and could potentially affect anemia tolerance [51]. For example, sepsis is a common condition in ICU. Due to the altered capability of the organism to utilize oxygen in this situation, whether the limit of anemia for oxygen transport changes in septic patients has been discussed. Holst and colleagues showed that a restrictive transfusion regime is not inferior compared to a liberal transfusion regime in septic shock patients [52]. Even if this does not demonstrate the physiological limits of acute anemia, it has been proven that the recently advised clinical approach (transfusion threshold 7 g/dL) is safe even in sepsis.

In most studies, it was possible to significantly reduce the perioperative transfusion requirements by a restrictive transfusion regime, which was associated with significant cost savings [53,54]. In a clinical situation with similar liberal and restrictive transfusion outcomes, the more cost-effective one is advised. 

Two important studies differ from this general finding that restrictive or liberal transfusion does not affect the outcome. First, Bergamin et al. demonstrated that a liberal transfusion regime was superior to a restrictive one in patients undergoing abdominal surgery due to an underlying malignant disease [55]. In contrast, in the study by Villanueva et al., mortality was decreased by a restrictive transfusion regimen in patients with gastrointestinal bleeding [56]. It is impossible to say whether these studies have extraordinary results due to the respective diseases or if should they be evaluated as random statistical errors (since both studies have methodological limitations). Overall, it can be stated that a clear superiority of one transfusion regime over another in terms of morbidity and mortality cannot be demonstrated. If one also considers the effort required to provide RBCs and the “primum nil nocere” principle, then a liberal transfusion regime can hardly be justified.

This general recommendation for a restrictive over a liberal transfusion regime has, of course, to be seen in the light of many comorbidities potentially influencing anemia tolerance in a specific patient. As long as this evidence has not been generated, existing meta-analyses can be interpreted in such a way that, so far, no comorbidity could be identified, which advises higher transfusion thresholds. An overview of this topic is provided by the latest Cochrane analysis by Carson and colleagues [47]. 

We know from the data presented that perioperative anemia is associated with increased perioperative morbidity and mortality. At the same time, it has been convincingly shown that unnecessary transfusion of RBCs does not improve clinical outcomes. For this reason, all available therapeutic measures to avoid anemia and transfusion of allogeneic blood must be utilized to improve outcomes. This leads to the third approach, the perspective of “Patient Blood Management” (PBM).

## 4. The PBM Point of View

Neither the acceptance of acute anemia nor the transfusion of RBC concentrates represents an effective therapeutic modality. Therefore, it seems logical that in daily clinical practice, everything must be undertaken to avoid both. This is the intellectual basis for a treatment concept that has gained significant traction in recent years, the so-called “Patient Blood Management” (PBM) [57].

**Figure 2:**
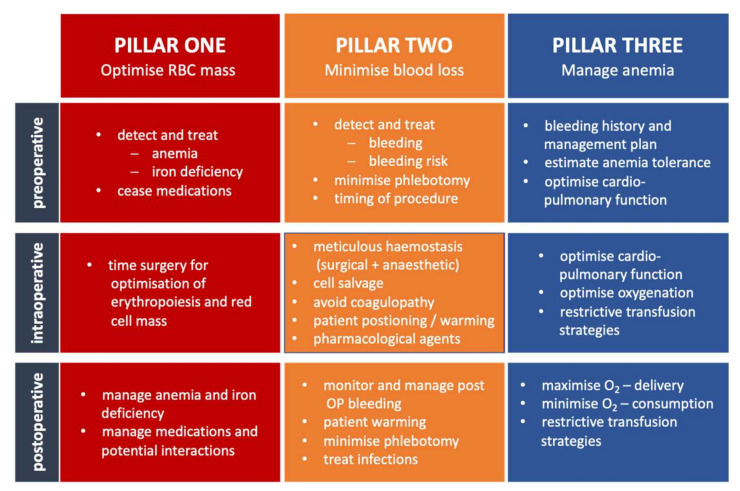
The “three pillar model” of PBM. Each pillar is divided into the pre-, intra-, and post-operative phase. Adapted from [58].

**Figure 3:**
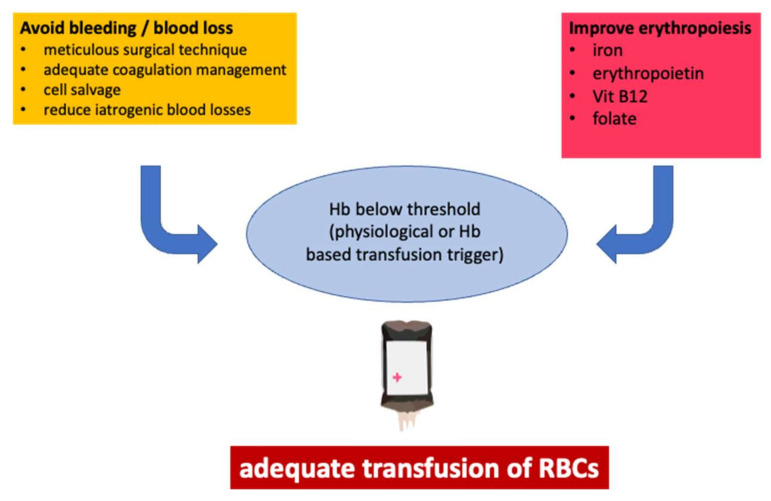
Usually, PBM is depicted as a three-pillar model, which initiates the idea that all three pillars are equally important. However, there is a clear hierarchy in single measures. The avoidance of bleeding and the improvement of erythropoiesis might help, but the Hb concentration remains high, and the transfusion of RBCs does not even have to be considered. However, adequate transfusion can be the answer if Hb falls below a certain threshold.

PBM aims to improve the quality of care through a clinical focus on increased erythropoiesis, reduced blood loss, and reflected transfusion (Figure 2) [58]. PBM is not a single measure, but a bundle of actions adapted depending on the individual patient [59]. These measures do not correspond to a “one size fits all” approach but must be individually put together for the particular clinical situation. It is essential to understand that in clinical practice, it is best to avoid the physiological or clinical limits of acute anemia. Preventing blood loss (the so-called 2nd pillar of PBM) or administering iron and erythropoietin to increase erythropoiesis (so-called 1st pillar of PBM) makes the transfusion of allogeneic blood unnecessary [60,61]. This approach is based on the hypothesis that moderate or even mild anemia is associated with worsening perioperative morbidity and mortality. The 3rd pillar of the PBM deals with the question of which threshold of Hb RBCs must be transfused in certain physiological situations (hypotension, hypoxia, sepsis, etc.) to prevent damage to the organism. Only this 3rd pillar of PBM defines the possibilities to exploit the physiological anemia tolerance in terms of a lower Hb or oxygen transport value. However, this does not mean that anemia should be tolerated until a Hb threshold for transfusion is reached, but that every form of anemia should be avoided as far as possible (Figure 3).

If therapy is needed, it should not be carried out by transfusion of erythrocyte concentrates but it should consider alternative treatment modalities for the individual case [62]. These treatment modalities include the administration of iron, erythropoietin, folic acid, and vitamin B_12_, which prove effective, even if applied in a concise time frame [63]. Which therapeutic option plays the most crucial role at a specific time is determined by several guidelines that refer to different clinical situations [64,65,66,67,68]. Despite the general belief that PBM is also very useful in ICU patients, and the fact that this has been demonstrated recently by Lasocki and coworkers [69], whether the application of iron at the ICU can increase the risk of nosocomial infection has been regularly discussed [70,71]; however, up to now, there has been no conclusive study that could prove this.

The efficacy of the PBM bundle was most impressively demonstrated in a large prospective observational study in Western Australia [53]. The interdisciplinary team reduced the odds ratio for mortality by 28% by implementing PBM and significantly reduced the transfusion of RBCs, fresh frozen plasma (FFP), and platelets, making PBM the standard for managing anemia, bleeding, and transfusion in Western Australia. A similar result was shown by Meybohm et al., where the implementation of PBM reduced the volume of blood products transfused [72]. However, no evidence regarding mortality could be demonstrated in this publication.

Every effort must be made in clinical practice to avoid anemia or treat it with adequate therapeutic modalities. Although the clinical approach commonly used in PBM has been successfully proven in large observational studies, there is some criticism about proving the effectiveness of individual interventions. However, whether this type of criticism is justified for a bundle of measures is still open for discussion [73]. In particular, the application of iron and erythropoietin is sometimes considered unsafe for some clinical situations such as acute infection for iron [74] and malignancy for erythropoietin [75]. So far, no randomized clinical trials exist that prove that the combination of a restrictive transfusion regime and postoperative application of iron and erythropoietin is superior to the transfusion of pRBCs. 

From what has been said so far, it can be concluded that two different limits to acute anemia exist. First, there is the naturally existing short-term anemia tolerance of the organism, which is defined by physiological conditions. The Hb values associated with this kind of anemia tolerance are low. Second, there is the tolerance of physicians to acute anemia, which is significantly higher but also associated with relevant morbidity and mortality. Both limits seem arbitrary for this reason because neither one of these limits is really “safe” from a clinical point of view. 

## 5. Conclusions

The concept of anemia tolerance is widely discussed in the recently published literature. However, this term subsumes very different clinical circumstances. Mainly, it stands for the tolerance of the organism to withstand acute, normovolemic anemia, but it also includes the phenomenon that both mild and moderate anemia are independent long-term risk factors for morbidity and mortality, and therefore should be avoided or treated. Correcting mild or moderate anemia with the transfusion of RBC concentrates rarely improves outcomes in a general population and should, therefore, only be used in specific indications. As a promising alternative, PBM includes a powerful toolbox to prevent and treat anemia.

## Data Availability

Not applicable.
